# Characterization of Peripheral Activity States and Cortical Local Field Potentials of Mice in an Elevated Plus Maze Test

**DOI:** 10.3389/fnbeh.2018.00062

**Published:** 2018-04-03

**Authors:** Toya Okonogi, Ryota Nakayama, Takuya Sasaki, Yuji Ikegaya

**Affiliations:** ^1^Laboratory of Chemical Pharmacology, Graduate School of Pharmaceutical Sciences, The University of Tokyo, Tokyo, Japan; ^2^Precursory Research for Embryonic Science and Technology, Japan Science and Technology Agency, Kawaguchi, Japan; ^3^Center for Information and Neural Networks, Suita City, Japan

**Keywords:** elevated plus maze, heart rate, hippocampus, neocortex, local field potential

## Abstract

Elevated plus maze (EPM) tests have been used to assess animal anxiety levels. Little information is known regarding how physiological activity patterns of the brain-body system are altered during EPM tests. Herein, we monitored cortical local field potentials (LFPs), electrocardiograms (ECGs), electromyograms (EMGs), and respiratory signals in individual mice that were repeatedly exposed to EPM tests. On average, mouse heart rates were higher in open arms. In closed arms, the mice occasionally showed decreased heart and respiratory rates lasting for several seconds or minutes, characterized as low-peripheral activity states of peripheral signals. The low-activity states were observed only when the animals were in closed arms, and the frequencies of the states increased as the testing days proceeded. During the low-activity states, the delta and theta powers of cortical LFPs were significantly increased and decreased, respectively. These results demonstrate that cortical oscillations crucially depend on whether an animal exhibits low-activity states in peripheral organs rather than the EPM arm in which the animal is located. These results suggest that combining behavioral tests with physiological makers enables a more accurate evaluation of rodent mental states.

## Introduction

Anxiety is a long-lasting state of apprehension caused by anticipating future threats and is linked with arousal, vigilance, and, in extreme cases, psychiatric disorders, such as post-traumatic stress disorder and social affective disorder. Conventional ideas assume that physiological manifestations of anxiety are represented by increased respiratory (Martin, 1961; Suess et al., 1980; Kim et al., 2013) and heart rates (Friedman and Thayer, 1998; Depino and Gross, 2007) and decreased locomotor activity.

**Figure 1:**
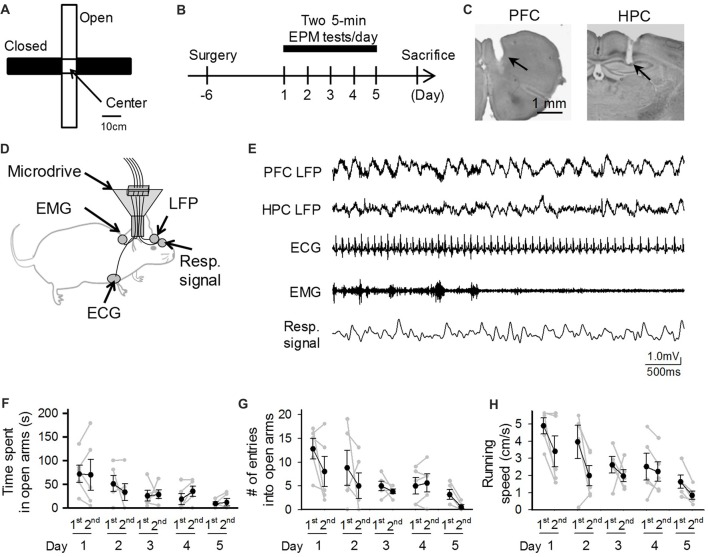
Behavioral performances of mice repeatedly exposed to elevated plus maze (EPM) tests. **(A)** An overview of the maze. **(B)** A mouse was subjected to EPM tests for five consecutive days. One recording day was composed of two 5-min sessions (1st and 2nd sessions) with an intersession interval of 10 min. **(C)** Histological confirmation of a recording site in the prefrontal cortex (PFC) and dorsal hippocampal CA1 (HPC) areas in a cresyl-stained section. The arrowheads indicate the electrode tracks. **(D)** Schematic illustration of the recording configuration from a freely moving mouse. **(E)** Representative electrophysiological recordings of local field potential (LFP) signals from the PFC and HPC, an electrocardiogram (ECG) signal, an electromyogram (EMG) signal from the dorsal neck muscle, and a Resp signal from the olfactory bulb. **(F–H)** Average percentage of time spent in open arms, number of entries into open arms, and running speed in the maze. Each gray dot represents one mouse. The statistical results in these figures are described in the main text.

In experiments using rodents, elevated plus maze (EPM) tests are one of the most widely used behavioral tests to assess innate anxiety-like behavior (Figure 1A; Handley and Mithani, 1984; Walf and Frye, 2007; Komada et al., 2008). In standard EPM test protocols, rodent animals are allowed to freely access two open and two closed elevated arms for several minutes. Based on the intrinsic characteristics of animals tending to avoid open arms because of higher levels of fear and anxiety, anxiety-related behavior is quantified by the total number of entries into the open/closed arms and the time spent in the open/closed arms. Ample evidence supports the EPM test as having the necessary face, construct and predictive validities to assess anxiety (File et al., 1994; Rodgers and Dalvi, 1997; Korte and De Boer, 2003; Walf and Frye, 2007).

While a number of neuroscience studies have been conducted utilizing EPM tests, little information on how physiological signals are dynamically altered and correlated with an animal behavioral performance during EPM tests is available. Given that anxiety intensity is altered depending on the arm type wherein an animal is located, one would expect to detect correlational changes between physiological markers related to anxiety and an animal’s behavioral performance in the maze.

To address this issue, we simultaneously monitored cortical local field potentials (LFPs), representing collective neuronal activity, electrocardiogram (ECG) signals, representing cardiac rhythm, electromyogram (EMG) signals, representing awake-related muscle contraction, and olfactory bulb LFPs, representing respiratory (Resp) signals, from a single mouse that was repeatedly subjected to identical EPM tests for 5 days (Figure 1B).

## Materials and Methods

### Approvals

This study was carried out in accordance with NIH guidelines for the care and use of animals. The protocol was approved by the Experimental Animal Ethics Committee of the University of Tokyo (approval number: P29-14).

### Subjects

A total of five male ICR mice (3–4 months old) with preoperative weights of 45–50 g were used in this study. The animals were housed individually and maintained on a 12-h light/12-h dark schedule with lights off at 7:00 AM. All animals were purchased from SLC (Shizuoka, Japan). After at least 1 week of adaptation to the laboratory, the mice underwent surgery.

### Surgery

Surgery was performed to implant electrodes for recording ECG, EMG, LFP, Resp signals in individual mice. The detailed surgical procedures have been described elsewhere (Okada et al., 2016; Sasaki et al., 2017). Briefly, before surgery, an electrode comprising a core body and a custom-made electrical interface board (EIB) accommodating four LFP channels, two ECG channels, one EMG channel, one Resp channel, and two ground/reference channels was assembled. For the surgery, mice were anesthetized with 1%–2% isoflurane gas in air. Two incisions (1 cm) were made on both sides of the upper chest, and two ECG electrodes (stainless steel wires; AS633, Cooner Wire Company) were sutured to tissue underneath the skin of the upper chest. The animal was then fixed in a stereotaxic instrument with two ear bars and a nose clamp. A midline incision was made from the area between the eyes to the incised neck area, and one EMG electrode (stainless steel wires; AS633, Cooner Wire Company) was sutured to the dorsal neck muscles. Circular craniotomies 1 mm in diameter were made using a high-speed drill at the following coordinates: 5.0 mm anterior and 0.5 mm unilateral to Bregma for the olfactory bulb, 1.7 mm anterior and 0.6–0.7 mm bilateral to Bregma for the prefrontal cortex (PFC), 2.0 mm posterior and 1.7 mm bilateral to Bregma for the hippocampus and 6.5 mm posterior and 1.5 mm bilateral to Bregma for the cerebellum. For the olfactory bulb and cerebellum craniotomies, stainless steel screws were implanted on the skull attached to the brain surface, serving as Resp electrodes and ground/reference electrodes, respectively. The open edges of all electrodes were soldered to the corresponding channels on the EIB. For the PFC craniotomy, an electrode was implanted at a depth of ~0.5 mm and an angle of 10 degrees. For the hippocampus craniotomy, an electrode was vertically implanted at a depth of ~1 mm. All wires and the microdrive were secured to the skull using dental cement. After completing all surgical procedures, anesthesia was removed, and the mice were allowed to awaken from the anesthesia spontaneously. Following surgery, each animal was housed in a transparent Plexiglas cage with free access to water and food.

### Elevated Plus Maze Task

The EPM was made of ABS resin and consisted of a central square (7.6 × 7.6 cm) and four arms (28 cm long × 7.6 cm wide, two open arms with no railing and two closed arms enclosed by a transverse wall 15 cm in height; Figure 1A). The maze was elevated 30 cm from the floor and illuminated with two 32-W fluorescent overhead lights, which produced light intensities of 250 ± 20 lux and 170 ± 20 lux in the open and closed arms, respectively. One recording day included two recording sessions with an intersession interval of 10 min (Figure 1B). In a recording session, a mouse was placed in the center of the central square facing the open arm and allowed to explore the maze apparatus for 5 min. After performing each session, the mouse was removed from the maze, placed into a transparent Plexiglas cage, and allowed to rest for 10 min; the floor of the field was cleaned with water and 70% ethanol during this period. The animal’s behavior in the maze was monitored at a frame rate of 60 Hz using a video camera, which was controlled by a laptop computer. In animal behavior analyses, the ratios of open or closed arm entries/time to the total arm entries/time were calculated. The frame rate of the movie was downsampled to 3 Hz, and the instantaneous speed of each frame was calculated based on the distance traveled within a frame (~333 ms).

### Electrophysiological Recording

Each mouse was connected to the recording equipment via Cereplex M (Blackrock), a digitally programmable amplifier, close to the animal’s head. The headstage output was conducted via a lightweight multiwire tether and a commutator to the Cereplex Direct recording system (Blackrock), a data acquisition system. LFP recordings were sampled at 2 kHz and filtered between 0.1 Hz and 500 Hz.

### Histological Analysis to Confirm Electrode Locations

The mice were overdosed with urethane/α-chloralose, perfused intracardially with 4% paraformaldehyde (PFA) in phosphate buffered saline (pH 7.4) and then decapitated. After dissection, the brains were fixed overnight in 4% PFA and then equilibrated with 30% sucrose in phosphate buffered saline. Frozen coronal sections (100 μm) were cut using a microtome, and serial sections were mounted and processed for cresyl violet staining. For cresyl violet staining, the slices were rinsed in water, counterstained with cresyl violet, and coverslipped with Permount. The positions of all electrodes were confirmed by identifying the corresponding electrode tracks in histological tissue.

### Data Analysis

All analyses were performed using Matlab (Mathworks). ECG traces were bandpass filtered at 20–200 Hz, and beat-to-beat intervals (R-R interval) were calculated from the timestamp of the R-wave peak. EMG traces were converted to root mean square (RMS) traces with a bin size of 500 ms every frame (~333 ms), which represents absolute changes in EMG amplitude relative to the baseline values. Resp and LFP signals were convolved by a Morlet’s wavelet family. In the power spectrum of Resp signals, maximum peaks were automatically detected, and instantaneous breathing rates were estimated based on the detected peaks. In each 5-min session in each animal, candidate periods with low peripheral (LP) states were detected when: (1) the ratio of 2–4 Hz power to 4–10 Hz power of the Resp signal was greater than 2; and (2) the heart rate was below the top 10% heart rate observed within the 5-min session. The first criterion was set to detect stable respiratory cycles without sniffing behavior and the second criterion was set to exclude apparently high instantaneous heart rates (the top 10%) due to an intense movement or false positive signals of heartbeats, if any. The heart rates at the second criterion were 727.7 ± 5.6 bpm (beats per minute; *n* = 50 sessions from 5 animals). LP states were defined for periods lasting at least 5 s. All data are presented as the mean ± standard error of the mean (SEM). The null hypothesis was rejected at the *P* < 0.05 level unless otherwise specified.

## Results

### Behavioral Performances of Mice Implanted With Electrodes in EPM Tests

We implanted electrodes in appropriate brain and body locations to simultaneously capture bioelectrical signals, including LFPs from the PFC and hippocampal CA1 (HPC) regions, as well as ECG, EMG and Resp signals (Figures 1C,D). All these systemic signals were integrated onto an amplifier on the mouse’s head. Figure 1E shows a representative recording of body-brain bioelectrical signals from a freely moving mouse. In total, five mice were subjected to EPM tests for five consecutive days, termed day 1 through day 5, with each recording day consisting of two 5-min EPM tests (Figure 1B). We first quantified the behavioral performances of these electrode-implanted mice in EPM tests. In each session and each mouse, the time spent in open arms and the number of entries into open arms in a 5-min test session were calculated (Figures 1F,G). On the first (1st) session of the first day (Day 1), the percentage of time spent in open arms was 24.1%, ranging from 0% to 57.6%, showing that the mice with electrode implantation stayed in the open arms for a lesser amount of time compared to that in closed arms. Time spent in open arms tended to, but not significantly (*F*_(4,49)_ = 2.329, *P* = 0.10, repeated-measures ANOVA), decrease as the testing days progressed and differed significantly between day 1 (first day) and day 5 (last day; Figure 1F; *t*_(9)_ = 2.97, *P* = 0.016), whereas both the number of entries into open arms and the running speed decreased as the testing days progressed (Figure 1G: number of entries into open arms: *F*_(4,49)_ = 3.53, *P* = 0.030; Figure 1H: running speed: *F*_(4,49)_ = 5.25, *P* = 0.0070, repeated measures ANOVA). Overall, these behavioral patterns are consistent with those reported in naïve mice that were subjected to repeated EPM tests (File et al., 1990; Schneider et al., 2011; Tucker and McCabe, 2017).

### Physiological Signals Differ Between Open and Closed Arms

**Figure 2:**
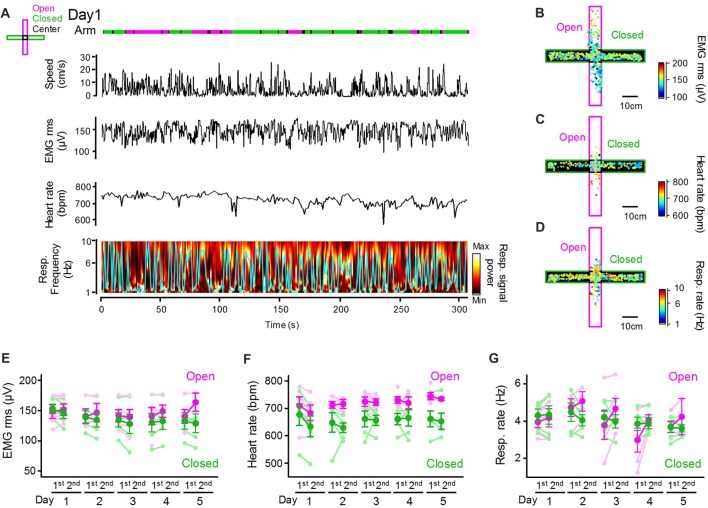
Peripheral physiological signals during the EPM test. **(A)** Representative animal’s running speed, root mean square (RMS) of an EMG signal, heart rates and color-coded power spectrum of a respiratory signal with the frequency giving the maximum power indicated by the cyan line. Periods when the animal was located in open and closed arms and the center are represented in magenta, green and black lines, respectively. **(B–D)** Color-coded animal’s trajectory shown by dots. The color codes represent the RMS of EMG signals **(B)**, heart rates **(C)** and respiratory rates **(D)** corresponding with an individual animal’s locations. **(E–G)** Average RMS of EMG signals, heart rates and respiratory rates plotted for open and closed arms in individual sessions. The statistical results in these figures are described in the main text.

Next, we assessed how peripheral physiological signals are altered with changes in behavioral patterns in the EPM. An example dataset depicting an animal’s positions and running speed and a series of peripheral activity patterns converted from individual bioelectrical signals is shown in Figure 2A. The amplitude of EMG signals was computed as the RMS, representing an animal’s movement and arousal state. Instantaneous heart rates were computed by averaging the individual RR intervals of ECG signals. Instantaneous respiratory rates were computed as frequencies giving the maximum power in the power spectrum of the Resp. signals (as represented by the cyan line) in a 1-s bin. Though the olfactory bulb signal does not perfectly reflect breathing cycles, Resp. signals at least partially represent relative changes in respiratory rates, including the stable respiratory rhythm at 2–4 Hz and sniffing behavior with a frequency of more than 5 Hz (Wesson, 2013; Tsanov et al., 2014; Sasaki et al., 2017). Representative color-coded maps were constructed showing how the amplitudes of EMG signals (Figure 2B), heart rates (Figure 2C), and respiratory rates (Figure 2D) in an animal were altered corresponding with its positions in the maze. These physiological indices were averaged across animals for each arm type as shown in Figures 2E–G. On average, the heart rates in closed arms were significantly lower than those in open arms (Figure 2F; *F*_(1,73)_ = 28.83, *P* = 3.4 × 10^−7^; three-way ANOVA (arm × trial × day)), consistent with the hypothesis that heart rates reflect animal anxiety levels and become lower in closed environments when anxiety levels are lowered. On the other hand, no significant differences in the RMS of EMG signals or respiratory rates between the open and closed arms were observed (Figure 2E; RMS of EMG signals: *F*_(1,73)_ = 3.17, *P* = 0.079; Figure 2G; respiratory rate: *F*_(1,73)_ = 0.22, *P* = 0.64; three-way ANOVA (arm × trial × day)).

### The Emergence of Low-Peripheral Activity States of Peripheral Physiological Activity Varies Across Days

**Figure 3:**
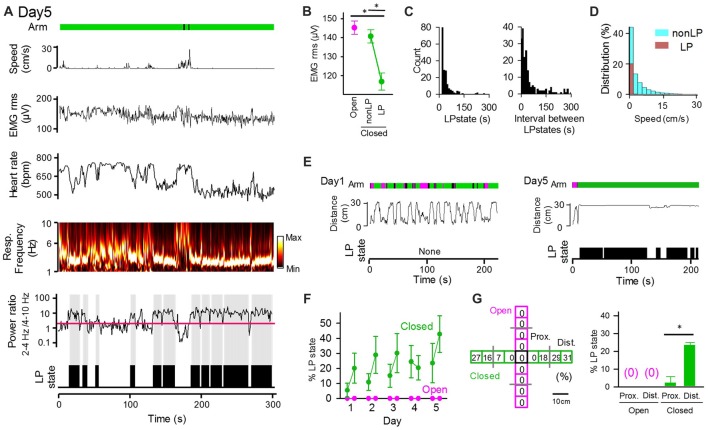
The low peripheral (LP) states of peripheral activity in closed arms vary across sessions and days. **(A)** The same representative data as that shown in Figure 2A. The ratio of 2–4 Hz power to 4–10 Hz power of respiratory signals is shown under the power spectrum, with the red horizontal line indicating a ratio of 2. LP states are represented by black areas below the traces. **(B)** RMS of EMG signals in LP states and non-LP states. *P < 0.001, paired *t*-test. **(C)** (Left) Distribution of the duration of single LP states. (Right) Distribution of the intervals of LP states. **(D)** Distribution of animal’s moving speed depicted separately in LP (brown) and non-LP states (cyan). **(E)** (From top to bottom) Arms wherein the animal was located at the same position as that in **(A)**, distance between the animal’s locations and the center of the maze, and periods of the LP states shown in **(A)**. **(F)** Average probability of LP states plotted for open and closed arms in individual sessions. The statistical results in these figures are described in the main text. **(G)** (Left) Each arm of the maze was divided into four regions, and the ratio of the duration of LP states to the total duration the animals spent in each region was calculated. (Right) Average probability of LP states plotted for regions wherein the animals were located. Proximal and distal regions were defined as regions with distances from the center of the maze less than and greater than 15 cm (the half length of an arms distance), respectively. *P < 0.001, paired *t*-test.

Statistical results from the averaged datasets showed a significant difference in only heart rates between open and closed arms (Figure 2F). However, pronounced fluctuations of these physiological measures were observed in closed arms even when the animals were located at nearly identical locations in the maze (Figures 2C,D). Specifically, we intermittently detected periods during which the animals exhibited reduced heart rates and maintained the maximum power of respiratory signals at the frequency of 2–4 Hz, which represents the basal respiratory rhythm when the mice are inactive without sniffing behavior (Figure 3A). Here, these states were defined as low-peripheral activity (LP) states, which were considered a possible marker of stabilized body states of the animals. The criteria of LP states were: (1) a ratio of 2–4 Hz power to 4–10 Hz power of respiratory signals greater than 2 (as represented by the red line in Figure 3A); (2) a heart rate below in the top 10% heart rates observed within the session; and (3) periods having these physiological activity patterns lasting for at least 5 s. LP states were not observed in open arms (Figure 3F). Periods with detectable LP states in closed arms had significantly lower EMG signal amplitudes than those in open arms and those without LP states (non-LP states) in closed arms (Figure 3B; open vs. non-LP states, *t*_(4)_ = 0.69, *P* = 0.28; open vs. LP states, *t*_(4)_ = 11.09, *P* = 6.6 × 10^−5^; non-LP vs. LP, *t*_(4)_ = 10.97, *P* = 7.0 × 10^−5^; paired *t*-test with Bonferroni correction). We analyzed the time and EPM locations that the animals exhibited LP states. As shown in the distribution in Figure 3C, single LP states lasted for 41.5 ± 2.9 s with a median of 28 s. The average time interval between two LP states was 95.4 ± 10.9 s with a median of 29 s. The distribution of animal’s moving speed in LP states and non-LP states is separately shown in Figure 3D. The distribution demonstrates that: (1) animals could exhibit LP states only when they completely stopped with a moving speed of 0–2 cm/s; and (2) even in periods during which a running speed was within a range of 0–2 cm/s, 31.5% of the periods included LP states, suggesting that stopping behavior only could not explain the occurrence of LP states. On day 1, an animal showed high locomotor activity throughout the maze, as represented by the frequent changes in the distance from the center of the maze, but had no LP states on day 1 (Figure 3E, left). On the other hand, the same animal showed little locomotor activity and exhibited apparent LP states in most periods when in closed arms on day 5 (Figure 3E, right). In all of the animals tested, no LP states were identified in animals located in open arms throughout the 5-day sessions (Figures 3F,G). The average percentage of periods with LP states in animals in closed arms was 5.5% on day 1 session 1 and gradually increased to 23.6% on day 5 as the testing days progressed. Taken together, these results demonstrate that not all periods in closed arms correspond to LP states, and the frequencies of LP states depend on how many times the mouse experiences the environment. We then analyzed whether the positions in the closed arms affect the emergence of LP states (Figure 3G). The probability of detecting LP states became higher when the animals were located in the distal part of the closed arms. Statistically, the percentage of LP states in the distal half of the closed arms was significantly higher than that in the proximal half (*t*_(48)_ = 5.82, *P* = 8.3 × 10^−7^, paired *t*-test). This result is consistent with the general idea that mice tend to have less anxiety in more closed environments.

### Cortical LFP Signals Depend on Peripheral LP States Rather Than Arm Type

**Figure 4:**
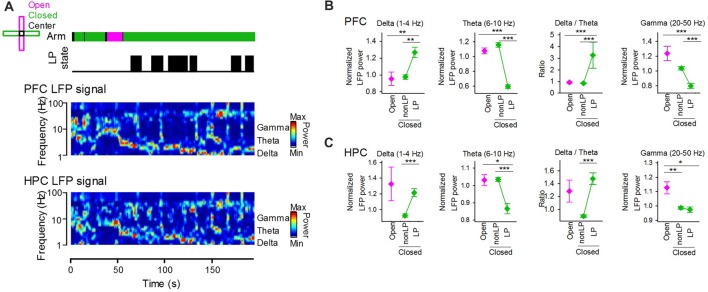
**(A)** (From top to bottom) Arms wherein the animal was located, periods of LP states and LFP signals from the hippocampus and frontal cortex. The color-coded power spectrum constructed from the LFP signals. **(B)** Average powers of PFC LFP signals at the delta, theta and gamma bands in periods in which the animals were located in open and closed arms. The closed arms were further divided into two periods with and without LP states (LP and non-LP, respectively). At each frequency band, the LFP power was normalized by that averaged over the entire period of each recording session. The right panel shows the ratio of delta power to theta power. **P < 0.01, ***P < 0.001, Wilcoxon signed-rank test with Bonferroni correction. **(C)** Hippocampal LFP signals at individual frequency bands. Like in **(B)**, data for the hippocampal LFP delta, theta, gamma powers are plotted. *P < 0.05, **P < 0.01, ***P < 0.001, Wilcoxon signed-rank test with Bonferroni correction.

We tested how the emergence of peripheral LP states is related to cortical LFP signals. Figure 4A shows an example dataset that includes the peripheral LP state periods and the power spectrum of LFP signals recorded from the PFC and HPC. LFP power was separately quantified based on arms and the presence of LP states (Figures 4B,C). At delta, theta, gamma bands, no significant differences in the LFP power of PFC LFP signals between when animals were in open arms and closed arms without LP states were observed (Figure 4B; open vs. closed non-LP; delta, *Z* = 0.89, *P* = 0.81; theta, *Z* = 2.01, *P* = 0.16, gamma, *Z* = −2.01, *P* = 0.16, Wilcoxon signed-rank test with Bonferroni correction). On the other hand, a significant increase and a significant decrease in LFP power at the delta and theta bands, respectively, between animals in closed arms with and without LP states were observed (closed non-LP vs. closed LP; delta, *Z* = 3.10, *P* = 0.010; theta, *Z* = −5.23, *P* = 1.5 × 10^−6^, Wilcoxon signed-rank test with Bonferroni correction), leading to a significant increase in the ratio of delta power to theta power in the presence of LP states in closed arms compared with that in the absence of LP states in closed arms (*Z* = 5.21, *P* = 1.5 × 10^−6^, Wilcoxon signed-rank test with Bonferroni correction). Similar to the frontal cortex, the theta power of hippocampal LFP signals was significantly decreased when the animals were in closed arms with LP states (Figure 4C left; open vs. closed non-LP, *Z* = 0.39, *P* > 0.99; closed non-LP vs. closed LP, *Z* = −4.10, *P* = 2.7 × 10^−4^, Wilcoxon signed-rank test with Bonferroni correction). These results demonstrate that the power of cortical LFP signals at delta and theta bands crucially depends on whether the animal shows peripheral LP states rather than on the EPM arm in which the animal is located. The gamma power of hippocampal LFP signals was significantly lowered in closed arms (open vs. closed non-LP, *Z* = −3.15, *P* = 0.0084; open vs. closed LP, *Z* = −2.60, *P* = 0.041, Wilcoxon signed-rank test with Bonferroni correction).

## Discussion

In this study, we monitored electrophysiological cortical LFP, ECG and EMG signals from individual mice that were repeatedly exposed to EPM tests in identical conditions. At the behavioral level, the number of entries into open arms and locomotor activity gradually decreased with a greater likelihood of staying in the closed arms as the testing proceeded for 5 days, which is consistent with the behavioral patterns of naïve mice that were repeatedly exposed to conventional EPM tests (File et al., 1990; Schneider et al., 2011; Tucker and McCabe, 2017). The mice implanted with a microdrive in the heads showed similar behavioral performances on the EPM tests and became less motivated to explore the open environments after repeated exposure to the maze. We found that the physiological brain and peripheral organ signals in these mice varied considerably across testing days even when the locations and moving speed of the mice were nearly identical in the same arms.

Irrespective of the testing day, heart rates averaged over the entire recording period were higher in open arms. This observation is consistent with the hypothesis that the levels of anxiety and/or motivation for exploration are presumably increased in open arms compared with that in closed arms, leading to increased cardiac activity. However, the frequencies of peripheral activity rhythms, including heart and respiratory rates, were not perfectly correlated with the animals’ locations in the EPM. Specifically, animals in closed arms occasionally exhibited a physiological state in which their instantaneous heart rates were lowered and their respiratory rate was stabilized at a frequency of 2–4 Hz, termed an LP state. LP states were never observed in open arms and they corresponded to periods of decreased EMG signals and no sniffing behavior with a respiratory rate of ~10 Hz. In most standard behavioral protocols, rodents are subjected to EPM tests only one time, which corresponds to the 1st period on the first day in our data. In this session, LP states were rarely (less than 10%) observed in closed arms. When repeatedly tested, LP periods were more frequently observed on the second test in a day or on latter days. Assuming that LP states represent periods when animal’s emotional excitability is reduced, the results suggest that the animal’s emotional states become less excited and more stabilized as the days of exposure to EPM tests increased. This possibility is consistent with the idea that LP states are more likely to appear after animals become familiar with the environment and/or are less motivated for exploration (File et al., 1990; Schneider et al., 2011; Tucker and McCabe, 2017). Also, our results confirm a general experimental rule that the day of testing (i.e., how many times animals experienced EPM test) should be considered for interpreting the data from EPM tests.

Notably, we found that LP states appeared only at distal regions of closed arms, whereas no such states were observed at regions close to the open center in the close arms. This result suggests that animal’s mental states are not similar but considerably vary across areas even in the same closed arms where anxiety levels have been uniformly quantified in previous behavioral studies based on the assumption that mental states are likely homogeneous in the same arms. Based on our data, we suggest that EPM data might be more accurately evaluated if the distance from the center of an EPM, not only which arms animals are located, is taken into consideration.

Cortical rhythmic activity is associated with the firing of single neurons with collective neuronal ensembles and facilitates information processing, including cognition, learning and memory (Buzsáki, 2006). In the neocortex, the delta and delta/theta power ratios of neocortical LFP signals, which have been used to define animal arousal levels (Vyazovskiy et al., 2009; Watson et al., 2016), were significantly increased only when the animals exhibited LP states in the closed arms. The amplitude of hippocampal theta oscillations, which correlates with an animal’s exploratory behavior and locomotion and are crucial for memory encoding and acquisition (Buzsáki, 2002; Colgin, 2016), was significantly decreased during the same period. These results together with the fact that no significant differences in the powers of these oscillations between open arms and closed arms without LP states were observed demonstrate that cortical LFP signals are more strongly correlated with the peripheral LP states of animals rather than with their absolute locations in the EPM. In addition, these results suggest that animals’ arousal levels, motivation for exploration, and even their anxiety intensity, might be similar between open and closed arms when they do not generate LP states.

EPM tests are widely used for assessing the effects of pharmacological drugs and genetic mutations on the anxiety levels of mice. However, the results of EPM tests are sometimes inconsistent with other behavioral tests of anxiety levels, such as a light/dark transition test (Holmes et al., 2003; Miyakawa et al., 2003; Hattori et al., 2012; Luckhart et al., 2016), possibly due to the fact that it includes panic-like behavior induced by higher anxiety, leading to longer stay periods in open arms in some circumstances. These difficulties in behavioral tests highlight a need to consider biomarkers to more accurately assess animal’s anxiety levels. In this respect, our results that brain physiological signals are determined by LP states even at the same location in EPM tests suggest that EPM data might be improved to more accurately evaluate anxiety levels when behavioral data are combined with physiological markers (Salas et al., 2003).

## Author Contributions

TO and TS designed the study. TO acquired the electrophysiological data. TO and RN performed the analyses. YI supervised the project. TO, RN and TS prepared all the figures. TS wrote the main manuscript text, and all the authors reviewed the main manuscript text.

## Conflict of Interest Statement

The authors declare that the research was conducted in the absence of any commercial or financial relationships that could be construed as a potential conflict of interest.
